# Delta-Sigma Modulated Visible Light Communication Illumination System Using a Projector with the Digital Micro-Mirror Device †

**DOI:** 10.3390/s19245512

**Published:** 2019-12-13

**Authors:** Motoi Kodama, Shinichiro Haruyama

**Affiliations:** Graduate School of System Design and Management, Keio University, 4-1-1 Hiyoshi, Kohoku-ku, Yokohama, Kanagawa 223-8526, Japan; haruyama@sdm.keio.ac.jp

**Keywords:** visible light communication, digital micro-mirror device, pulse width modulation, pulse density modulation, delta-sigma modulation, voice information guidance system

## Abstract

There is a unique transmission method of visible light communication (VLC) that can transmit multiple data in multiple directions simultaneously by using a projector with the digital micro-mirror device (DMD). Previously, we proposed a method of transmitting data from the projector that transmits digitally modulated VLC signal from each pixel, and in this paper, we propose an extension of the method to transmit audio signal by using a special type of modulation called delta-sigma-modulation (DSM). The DSM-VLC system employs a simple receiver that comprises simple analog electric circuits, which contribute to low power consumption. We made a DSM-VLC prototype and verified that the prototype was able to send four different waves to different directions. Additionally, the experiments’ results agree very well with the simulation results. Furthermore, we designed two types of DSM: pulse width modulation (PWM) and pulse density modulation (PDM), and we verified that the PDM-VLC is better than the PWM-VLC regarding the DMD switching frequency’s efficiency. Our proposed DSM-VLC can be used for such applications as voice information guidance systems with direction-selective messages.

## 1. Introduction

Visible light communication (VLC) is an optical wireless communication technique that utilizes the light visible to humans [1,2]. Since the VLC is safe to the human body even at high output and enables wideband communications, the VLC has attracted much attention due to its wide expanse of the light-emitting diode (LED) light and has various unique applications: intelligent transport systems [3], survey measurement system [4,5], robot control system [6,7,8], indoor navigation system [9], etc. The projector VLC (PVLC) has been studied to achieve the novel displays emitting imperceptible metadata along with the image [10,11,12,13,14,15]. For example, Kimura et al. developed EmiTable [12], which is a tabletop surface pervaded with imperceptible metadata, whereas Kato et al. developed iPvlc [13], which is pixel-level PVLC. Moreover, in these previously conducted studies, a projector with the digital micro-mirror device (DMD) could not be applied for the location service technology.

**Figure 1:**
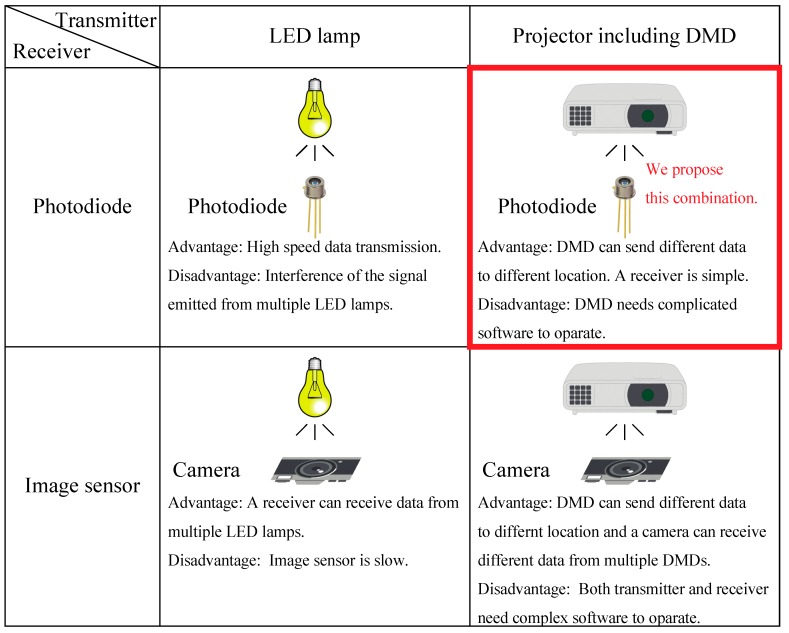
The transmitter and receiver in the conventional VLC and PVLC.

Figure 1 illustrates the transmitter and receiver in the conventional VLC and PVLC. The simplest VLC is a method with a LED lamp and a photodiode. Though its advantage is high speed data transmission, the interference of VLC signals emitted from multiple LED lamps occurs as its disadvantage. Generally, to solve such an interference issue and realize the spatial modulated VLC, an image sensor is used in substitution for the photodiode as the VLC receiver. The VLC using the LED lamp and the photodiode realizes the spatial modulated communication, but its speed is limited due to the image sensor’s frame rate. We paid attention to the PVLC to overcome such a problem. When a projector with the DMD is employed as the VLC transmitter, it can embed the different data for each pixel and modulate the VLC signals rapidly. Its disadvantage is that the DMD needs complicated software to operate it. Similarly, when the image sensor is employed as the VLC receiver, the image sensor needs complex software to operate it. So, the VLC using a projector with the DMD and a photodiode is the best solution for realizing the high speed and spatial modulated VLC in the circumstances.

**Figure 2:**
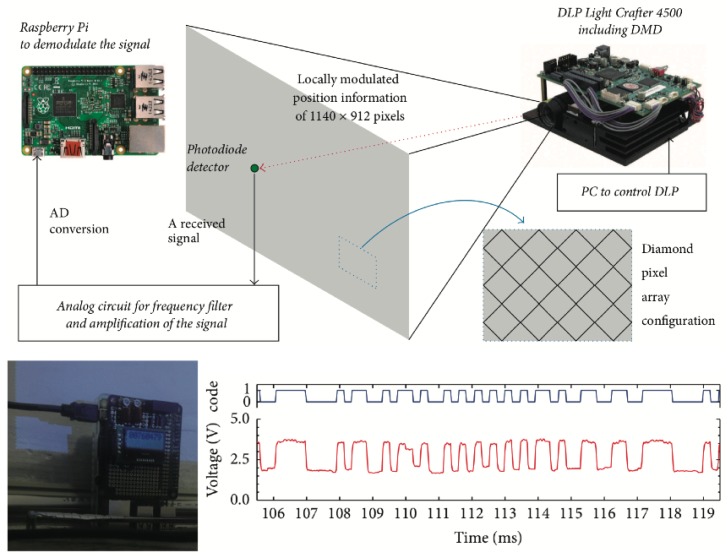
Our previously conducted experiments of a fine-grained VLC position detection illumination system with millimeter accuracy using the receiver with Raspberry Pi to demodulate the signals [18].

Our previously conducted studies [16,17,18] experimentally showed that a projector with the DMD as the PVLC’s transmitter could achieve more rapid and spatial modulated PVLC illumination-like location beacon. Figure 2 shows our previously conducted experiments of a fine-grained VLC position detection illumination system with millimeter accuracy using the receiver with Raspberry Pi to demodulate the signals [18]. When we consider new lighting technical method, it is important for its users not to feel its flicker noise, which can be eliminated at more than about 600 bps in 2 pulse width modulation (2PPM) [19,20]. Since a data rate in our proposed VLC position detection illumination system was about 2 kbps in the 2PPM, it keeps the users not being able to feel the flicker noise [16,17,18]. This previously conducted study was the first to utilize a projector with the DMD as an illumination, and we demonstrated that a projector with the DMD could experimentally achieve spatial modulated VLC illumination-like beacon in the Internet-of-Things (IoT) age [16,17,18].

Here, a much simpler position-dependent PVLC illumination system has been designed without requiring any data processing from a microcomputer. Table 1 shows the data form when the DMD and a photodiode are used in the PVLC. In our previously conducted studies [16,17,18], though its advantage is that a receiver can detect the receiver’s location with a few millimeters, there is its disadvantage that power consumption of a digital circuit is comparatively high. Hence, although non-audio data cannot be received, we focused on the digital audio technology to realize lower power consumption and a more suitable method for audio headphone. As our fundamental discussion, we reported the pulse width-modulated VLC (PWM-VLC) experiments in a conference proceeding [21].

In this study, we extend the PWM-VLC technique to the delta-sigma-modulated VLC (DSM-VLC) technique including the pulse density-modulated VLC (PDM-VLC) technique, as well as propose the DSM-VLC technique using a projector with the DMD for the voice information guidance system and report its basic experiments. In general, based on the PWM or the PDM signals, the delta-sigma modulation (DSM) method is frequently applied in the digital audio technology, which demodulates the signals using an analog electric circuit with a lowpass filter [22,23,24,25]. To amplify and demodulate the signals as digital-to-analog conversion, the DSM-VLC system utilizes a very simple receiver that comprises analog electric circuits driven by dry batteries. Thus, it does not require the complex digital signal processing with the microcomputer.

**Figure 3:**
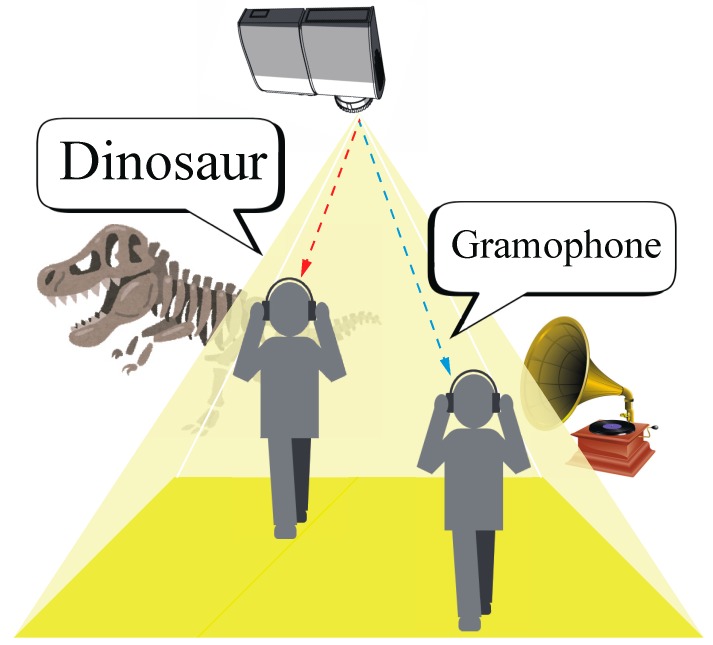
A use case of DSM-VLC using a projector with the DMD for voice information guidance illumination system in a museum [21].

Figure 3 illustrates a use case of DSM-VLC using a projector with the DMD for voice information guidance illumination system in a museum [21]. The museum visitors wear a headphone that includes the photodiode detector and analog electric circuits as the DSM-VLC receiver. When the museum visitors walk in front of exhibits that are lighted up by a PVLC illumination, their guidance is transmitted to the headphone as the DSM-VLC receiver. Besides, the headphones broadcast the sound that is not stored in any memory and the system only employs the real-time DSM-VLC method. As illustrated in Figure 3, to calculate the demodulated signals, the DSM-VLC receiver does not need to have any special device or the memory storage hardware to save the data such as the microcomputer. The user only requires a simple DSM-VLC receiver consisting of a photodiode detector, simple analog circuits, and a speaker. Thus, we would like to apply a system to such a fine-grained voice information guidance PVLC illumination system in the future. 

## 2. Materials and Methods

In this section, we introduced DSM, the experimental system and method for the DSM-VLC, the coding method for the PWM-VLC, the coding method for the PDM-VLC, and the simulation analysis method.

### 2.1. Introduction of DSM

**Figure 4:**
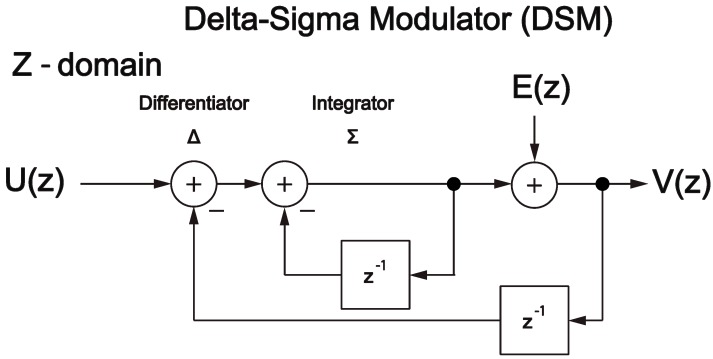
The DSM described in *Z*-domain [23].

We introduced DSM, which is the underlying principle of the DSM-VLC. Figure 4 depicts the delta-sigma modulator (DSM) described in Z-domain [25]. 

According to reference [25], in Z-domain model demonstrated in Figure 4, the relational expression between the input signal, U(z), and the output signal, V(z), is defined as follows:

(1) $$V\left(z\right)=U\left(z\right)+\left(1-z^{-1}\right)E\left(z\right),$$

where U(z) denotes the intended output signal component and $\left(1-z^{-1}\right)E\left(z\right)$ denotes the contribution of the quantization noise, which is the component of high frequency referred to as the noise shaping when compared with the frequency of U(z).

When demodulating from output signal, V(z), to input signal, U(z), the greatest advantage of the DSM is that the demodulated signal can be provided just to remove the noise shaping expressed as $\left(1-z^{-1}\right)E\left(z\right)$ by a low-path filter employed to perform the role of a digital-analog converter.

The DSM can also be called the sigma-delta modulation (SDM). In this study, we called this method the DSM regarding the opinion of the designers: Yasuda et al. [23,24,25].

### 2.2. Experimental System and Method for DSM-VLC

**Figure 5:**
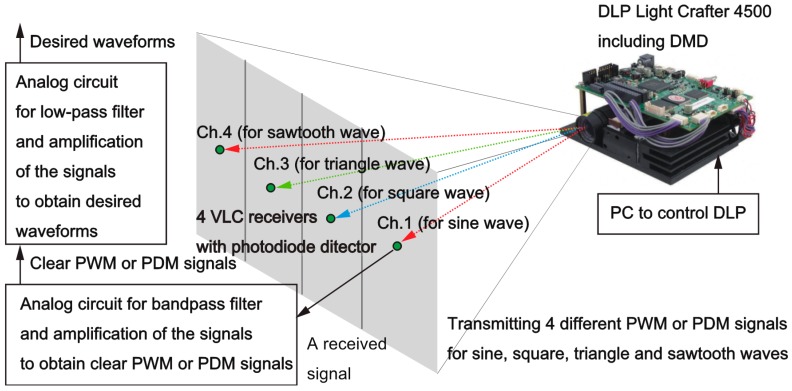
A schematic diagram of the DSM-VLC experimental system in our conducted experiments.

Figure 5 illustrates a schematic diagram of the DSM-VLC experimental system in our conducted experiments. As shown in Figure 5, we utilized the DSM-VLC system using a digital light processing (DLP) projector (DLP Light Crafter 4500 Evaluation Module, Texas Instruments Incorporated, Dallas, TX, USA), which has the DMD. The DSM-VLC system includes the DLP projector used as the DSM-VLC transmitter and four DSM-VLC receivers with photodiode detector that have analog circuits for the band-pass filter to eliminate the noise, which was caused by the room illumination, and obtain the clear PWM-VLC or PDM-VLC signals, as well as for the lowpass filter used as a digital-to-analog conversion’s element to obtain the desired waveform. In our DSM-VLC system prototype, a projector with the DMD transmits the following four types of waves concurrently: sine, square, triangle, and sawtooth waves. The DSM-VLC receivers receive each different wave’s signal.

**Figure 6:**
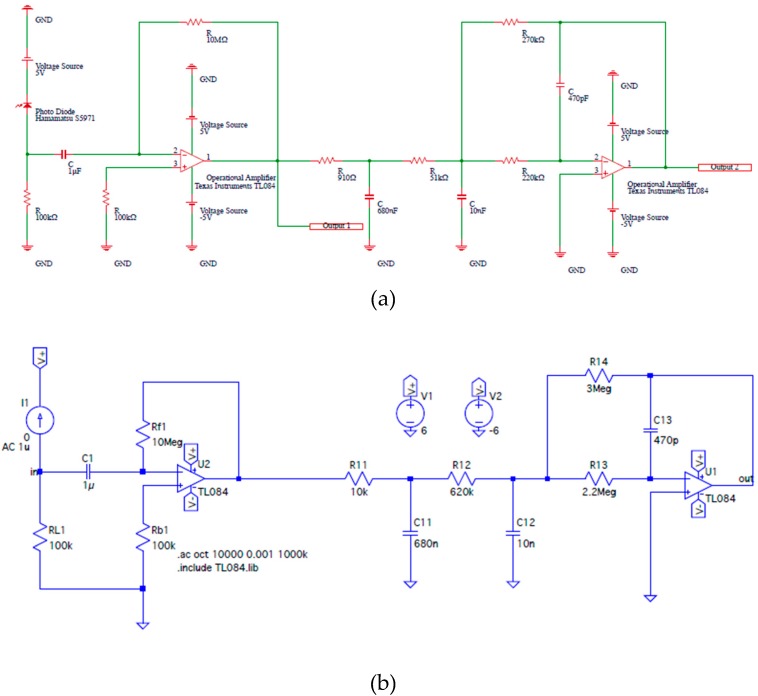

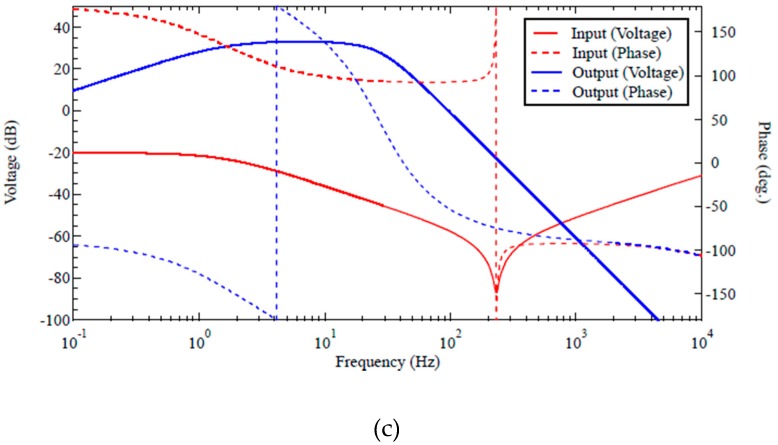
(**a**) A VLC receiver’s circuit diagram for the DSM-VLC experiments [21], (**b**) a circuit diagram for the simulation to design the VLC receiver’s circuit, and (**c**) the simulation results of the frequency characteristic of the input and output voltage in the circuit shown in (**b**).

Figure 6 shows a VLC receiver’s circuit diagram for the DSM-VLC experiments [21], (b) a circuit diagram for the simulation to design the VLC receiver’s circuit, and (c) the simulation results of the frequency characteristic of the input and output voltage in the circuit shown in (b). As demonstrated in Figure 6a, each DSM-VLC receiver comprises a photodiode detector, four dry batteries that drive the electric circuits, and two output signal’s Bayonet Neill–Concelman (BNC) connector to measure the inverted PWM-VLC or PDM-VLC signal, which is called “Output 1” and the demodulated four types of waves, which is called “Output 2.” Each electric circuit in the DSM-VLC receiver employs two operational amplifiers: one is used for the band-pass filter and the amplification, whereas the other is used for the lowpass filter as the digital-to-analog conversion component. Due to the performance limit of the DLP Light Crafter 4500 Evaluation Module in the PWM-VLC experiments, to compare the characteristics of PWM-VLC with those of PDM-VLC, we adopted that the frequency of four types of waves was 8.7 Hz in the DSM-VLC experiments. The cutoff frequency of the lowpass filter was 23 Hz, which was obtained by simulating the approximate circuit, which replaced the photodiode with an alternating current source whose amplitude was 1 μA as shown in Figure 6b. The filters’ frequency response characteristic was calculated as shown in Figure 6c, and we confirmed that there was no abnormal frequency response phenomenon like an oscillation in both circuits of Figure 6a,b.

**Figure 7:**
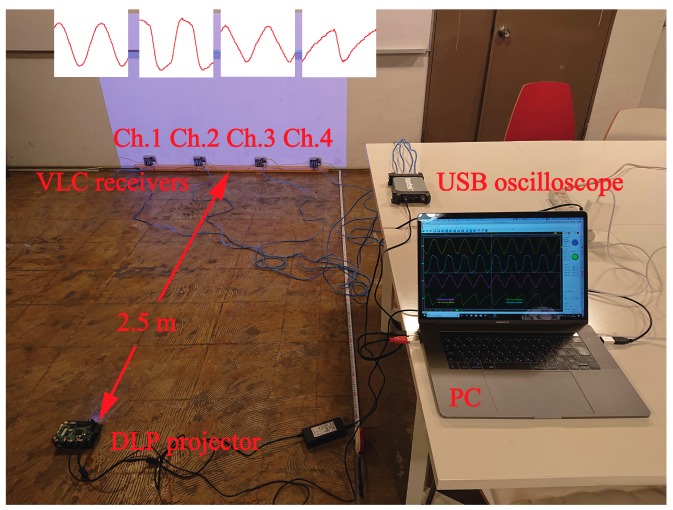
A photograph of the DSM-VLC experiments.

Figure 7 illustrates the photograph of the DSM-VLC experiments. The distance between the DLP projector and the DSM-VLC receiver was 2.5 m, which was the distance between the indoor ceiling attached to the light and the floor where the users exist. In the conducted experiments, there were four receivers to receive the four different PWM-VLC or PDM-VLC signals. Using a four-channel universal serial bus (USB) oscilloscope (Hantek 6254BD, Qingdao Hantek Electronic Co., Ltd., Qingdao, China) and a computer (MacBook Pro (15-inch, 2016), Apple Inc., Cupertino, CA, USA) operated under MacOS (Apple Inc., Cupertino, CA, USA), and Microsoft Windows 10 (Microsoft Corporation, Redmond, WA, USA) as MacOS’s virtual environment.) to control the DLP projector as well as the USB oscilloscope, we considered the PWM-VLC or PDM-VLC signals and the demodulated signals as the four DSM-VLC receiver’s output concurrently.

### 2.3. Coding Method for PWM-VLC

In the PWM-VLC experiments, due to the DMD performance limitation on the DLP Light Crafter 4500 Evaluation Module, we aimed to develop 16 sampling points’ waves in a period, and the frequency of wave was 8.728 Hz, which is a combination of wave’s frequency and the number of samplings to achieve a maximum DMD’s switching performance in the DLP Light Crafter 4500 Evaluation Module.

**Figure 8:**
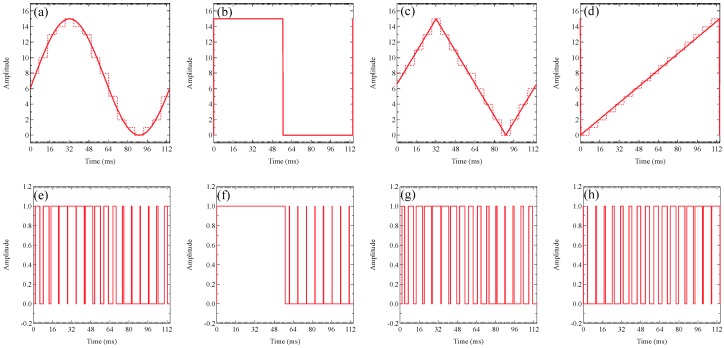
The original waveforms and 4-bit quantized amplitude’s PWM signals of (**a**) sine, (**b**) square, (**c**) triangle, and (**d**) sawtooth waves, and a single-bit PWM signal of (**e**) sine, (**f**) square, (**g**) triangle, and (**h**) sawtooth waves.

Figure 8 depicts the original waveforms and 4-bit quantized amplitude’s PWM signals of (a) sine, (b) square, (c) triangle, and (d) sawtooth waves, and a single-bit PWM signal of (e) sine, (f) square, (g) triangle, and (h) sawtooth waves. We adopted a 4-bit wave amplitude modulation and 16 sampling points in a period, and each wave was quantized by the wave such as the dotted lines demonstrated in Figure 8a–d. Since the minimum DMD’s switching period is 231 µs in the DLP Light Crafter 4500 Evaluation Module, the pulse width spreads on both sides by 231 µs to avoid the phase disorder of the demodulated wave. Therefore, one sampling time was 7.161 ms, i.e., the sampling frequency was 139.6 Hz.

### 2.4. Coding Method for PDM-VLC

In the PDM-VLC experiments, to compare the results of the PWM-VLC experiments and those of the PDM-VLC experiments, we aimed at developing 100 sampling points’ waves in a period, whose frequency is 8.728 Hz.

**Figure 9:**
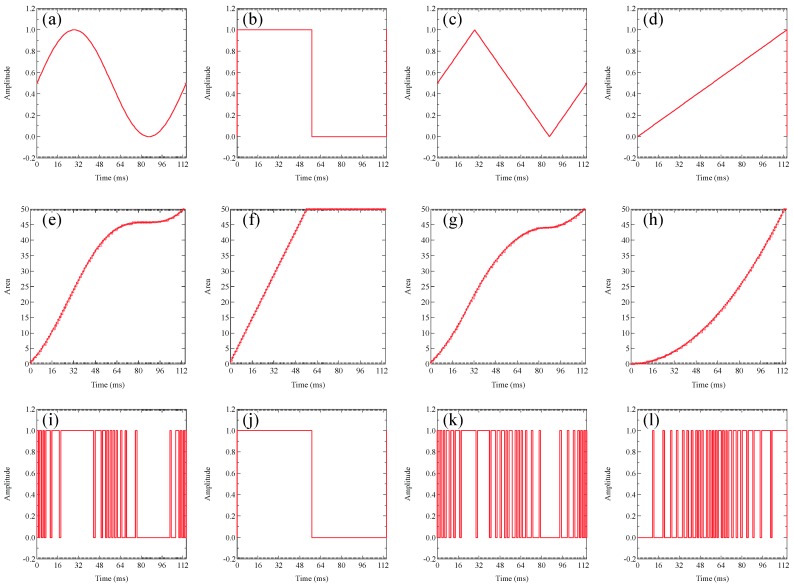
The original waveforms, a period’s area quantization, and a single-bit pulse-density modulated (PDM) signals of sine (**a**,**e**,**i**), square (**b**,**f**,**j**), triangle (**c**,**g**,**k**), and sawtooth (**d**,**h**,**l**) waves.

Figure 9 demonstrates that the original waveforms, a period’s area quantization, and a single-bit PDM signals of sine ((a), (e), and (i)), square ((b), (f), and (j)), triangle ((c), (g), and (k)), and sawtooth ((d), (h), and (l)) waves. Additionally, as shown in Figure 9e–h, we adopted the 50 levels area quantization and 100 sampling points in a period of each wave; when the quantized area of each wave increased by 1, the PDM signal of each wave stood up, as shown in Figure 9i–l.

When we utilized the DLP Light Crafter 4500 Evaluation Module to develop the wave with 100 sampling points in a period, whose frequency is 8.728 Hz, a sampling time became 1.146 ms, i.e., the sampling frequency became 872.8 Hz. This implies that the sampling frequency of the PDM-VLC was 6.252 times greater than that of the PWM-VLC, and the DMD’s switching frequency in the PDM-VLC was 4.960 times greater than that in the PWM-VLC.

### 2.5. Simulation Analysis Method

To compare the experimental results of the PWM-VLC with those of the PDM-VLC, we calculated the theoretical results using the LTspice (LTspice, Analog Devices, Inc., U.S.A.).

When the frequency of waves was 8.728 Hz, we simulated the input and output signals on the lowpass filter as a digital-to-analog conversion component.

Furthermore, when the frequency of the sine wave was 8.728 Hz in both the PWM-VLC and the PDM-VLC, we obtained the DMD switching frequency dependence of the total harmonic distortion (THD), which was calculated by the fundamental wave and five higher harmonic waves using MATLAB (MATLAB R2019b, The MathWorks, U.S.A.).

## 3. Results and Discussion

In this section, we reported the simulation and the experimental results, as well as discussed the characteristics of PWM-VLC and PDM-VLC.

**Figure 10:**
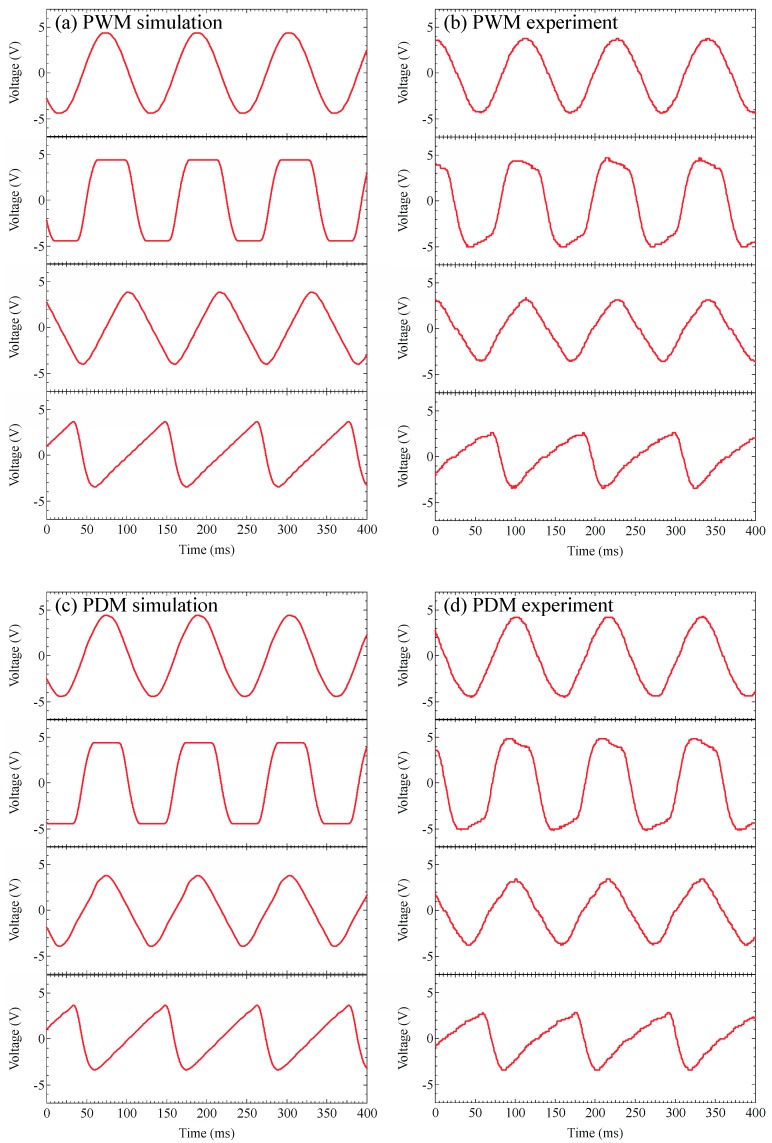
(**a**) The simulation results of the output PWM-VLC signals time dependence, (**b**) the experimental results of the output PWM-VLC signals time dependence, (**c**) the simulation results of the output PDM-VLC signals time dependence, and (**d**) the experimental results of the output PDM-VLC signals time dependence, in sine, square, triangle, and sawtooth waves.

Figure 10 illustrates (a) the simulation results of the output PWM-VLC signals time dependence, and (b) the experimental results of the output PWM-VLC signals time dependence, and (c) the simulation results of the output PDM-VLC signals time dependence, and (d) the experimental results of the output PDM-VLC signals time dependence, in sine, square, triangle, and sawtooth waves. As shown in Figure 10b, sine, square, triangle, and sawtooth waves with frequencies 8.741, 8.803, 8.741, and 8.621 Hz were rapidly demodulated in each domain, respectively. As shown in Figure 10d, sine, square, triangle, and sawtooth waves with frequencies 8.621, 8.621, 8.681, and 8.562 Hz were rapidly demodulated in each domain, respectively. As depicted in Figure 10b,d, square, triangle, and sawtooth waves’ corners were blunted as in the simulation results shown in Figure 10a,c. It seems to be the capacitive contribution in the receiver’s circuit filters’ effects.

**Figure 11:**
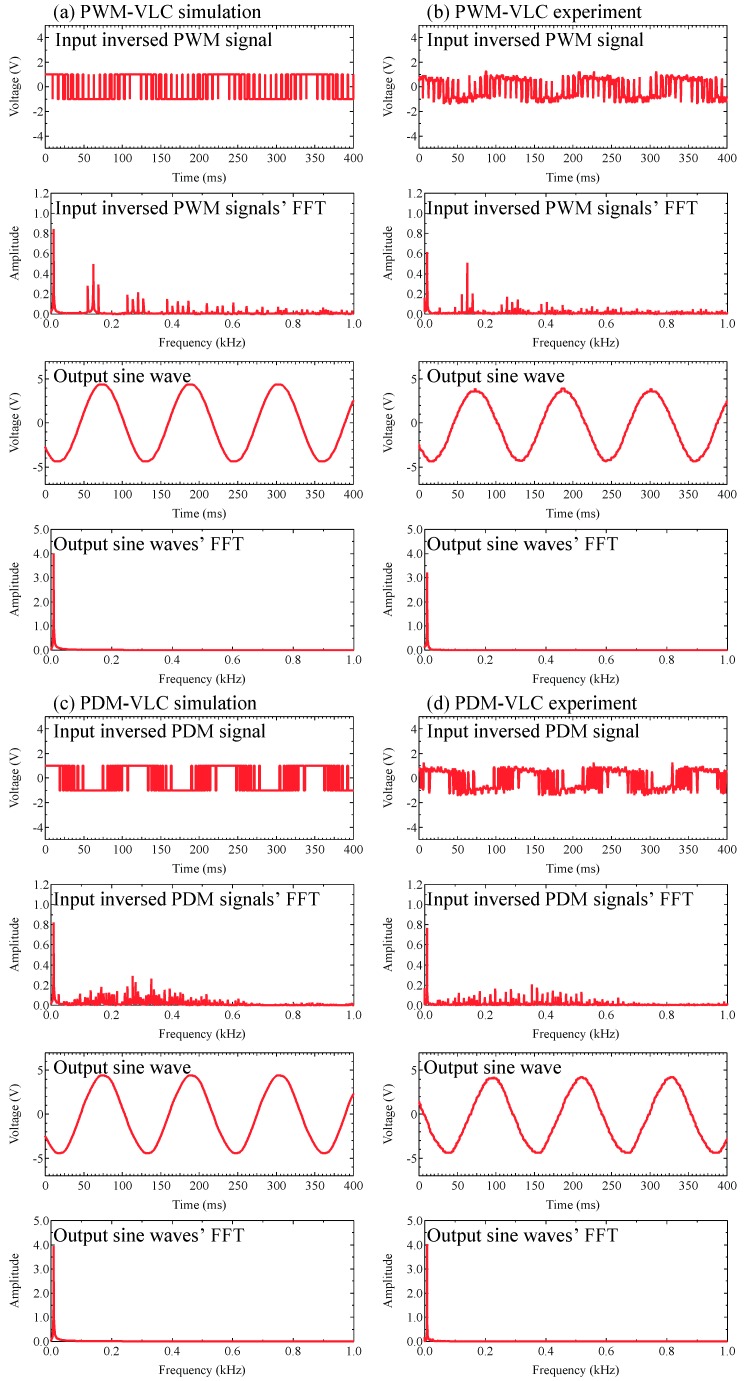
The input inverted modulated signals’ time dependence, its fast Fourier transform (FFT), output sine wave signals’ time dependence, and its FFT in (**a**) PWM-VLC simulation, (**b**) PWM-VLC experiment, (**c**) PDM-VLC simulation, and (**d**) PDM-VLC experiment.

Figure 11 shows the input inverted modulated signals’ time dependence, its fast Fourier transform (FFT), output sine wave signals’ time dependence and its FFT in (a) PWM-VLC simulation, (b) PWM-VLC experiment, (c) PDM-VLC simulation, and (d) PDM-VLC experiment.

Owing to the circuit’s configuration depicted in Figure 6a, the high and low states of input signals were inverted in the band-pass filter when compared with the PWM-VLC’s signals and the PDM-VLC signals, which mean the sine wave. Moreover, the pulse width of each of the sampling point’s signals did not change before and after going through the band-pass filter. As demonstrated in Figure 11, the lowpass filter used as a digital-to-analog conversion’s element generated the output signals demodulated as in the desired sine wave, and the PWM-VLC and PDM-VLC methods were achieved based on our theoretical design.

**Figure 12:**
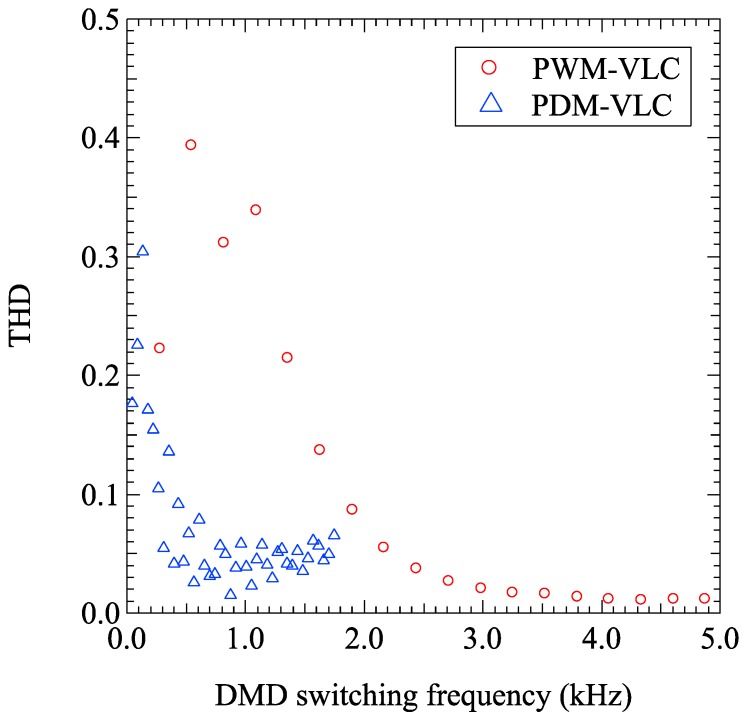
The simulation results of total harmonic distortion’s (THD’s) DMD switching frequency dependence when the frequency of sine waves made of the PWM-VLC and PDM-VLC is 8.7 Hz.

We calculated and discussed the total harmonic distortion (THD), which is the parameter of signal’s distortion to compare between the sine wave’s distortion in the PWM-VLC and that in the PDM-VLC. Figure 12 shows the simulation results of THD’s DMD switching frequency dependence when the frequency of sine waves made of the PWM-VLC and PDM-VLC is 8.728 Hz.

As shown in Figure 12’s red plots, the THD in the PWM-VLC becomes approximately constant 0.01 in more than the DMD switching frequency of about 3 kHz. According to the calculation of THD in the Figure 11, we chose the minimum THD, which was 0.0112 when the number of sampling was 16, i.e., the sampling frequency was 139.6 Hz whose DMD switching frequency was 4.329 kHz. From the PWM-VLC experimental result shown in Figure 10b, we obtained the THD of the sine as 0.0450 experimentally. As shown in Figure 12’s blue plots, the THD in the PDM-VLC became approximately constant 0.01 around the sampling frequency about 1 kHz. According to the calculation of THD in Figure 11, we chose the minimum THD, which was 0.0149 when the number of sampling was 100, i.e., the sampling frequency was 872.8 Hz, which was the DMD switching frequency. It was considered that the vibration of the THD levels depended on their quantization error due to the sampling number of times per one period of sine wave. 

From the PDM-VLC experimental result shown in Figure 11, we obtained the THD of the sine as 0.0446 experimentally. Since the amplitude of output sine wave pattern tends to become small in more than about 1.8 kHz and we could not evaluate them in the same standard, we did not plot them in Figure 11. We found out that the PDM-VLC’s THD was about 1/5 of PWM-VLC’s THD for the DMD switching frequency between 0 kHz and 1.5 kHz in calculational result, and the PDM-VLC was about five times better than PWM-VLC regarding the DMD switching frequency’s efficiency to create a sine wave with the same frequency at the same level’s THD in both calculational and experimental results.

Based on the results of the DSM-VLC experiments, we experimentally observed that the DSM-VLC system was feasible. According to the reference [26], typical values obtained for the frequency of the voice fundamental are 120 Hz for men and 210 Hz for women. Even if we employed DLP7000 XGA Chipset, which is the fastest available DLP, that can project a maximum of 32,552 Hz binary patterns [27] for DLP Discovery 4100, the frequency of wave only becomes 65.63 Hz in the case of 16 sampling points’ waves in a period, in the PWM-VLC method; it increases to 325.52 Hz in the case of 100 sampling points’ waves in a period, in the PDM-VLC method. In this technology of a projector with the DMD, although the PWM-VLC is not enough to transmit the human voice, we considered that the PDM-VLC could transmit the human voice that has a low frequency: a few hundred hertz. On the other hand, to realize the PDM-VLC illumination for voice information guidance system for its users not to feel flicker noise which can be eliminated at more than about 600 bps in 2 pulse width modulation (2PPM) [19,20], at least, the DMD’s switching speed should be about two times faster than DLP7000 XGA Chipset. By employing the DSM-VLC receiver and a projector with the DMD with millimeter accuracy in the future, a projector with the DMD that speeds up more allows the users to receive the high-resolution and high-frequency voice information guidance depending on their position.

## 4. Conclusions

In this study, we proposed the DSM-VLC, including the PWM-VLC and the PDM-VLC, using a projector with the DMD as the voice information guidance system, and we reported the fundamental simulations and experiments conducted. 

The DSM-VLC system employed a very simple DSM-VLC receiver that comprises of analog electric circuits to amplify and demodulate the signals as the digital-to-analog conversion. By applying the DSM-VLC technique, the conducted basic experiments showed that the DSM-VLC system was able to transmit different audio signals to different directions concurrently. The experimental results agreed very well with the simulation results.

Furthermore, based on the calculational and experimental results, we observed that the PDM-VLC’s THD was about 1/5 of PWM-VLC’s THD for the DMD switching frequency between 0 kHz and 1.5 kHz, and the PDM-VLC was about five times better than PWM-VLC regarding the DMD switching frequency’s efficiency to create a sine wave with the same frequency at the same level’s THD.

By utilizing the DSM-VLC receiver and a projector with the DMD as the DSM-VLC transmitter with millimeter position accuracy, the projector whose DMD’s switching speed was about two times faster than that of DLP7000 XGA Chipset would allow the users to receive the location-dependent voice information guidance message without feeling the flicker noise caused by PVLC’s illumination.

## Figures and Tables

**Table 1:** The data form when the DMD and a photodiode are used in the VLC.

	Any Digital Data [16,17,18]	Digital Audio Data [21]
Advantage	A receiver can detect the receiver location.	Low power consumption. Suitable for audio headphone.
Disadvantage	Power consumption of a digital circuit is comparatively high.	It cannot receive non-audio data.

## References

[1] Tsonev D., Videv S., Haas H. Light fidelity (Li-Fi): Towards alloptical networking. Proceedings of the SPIE 9007, Broadband Access Communication Technologies VIII, 900702.

[2] Kumar A., Mihovska A., Kyriazakos S., Prasad R. (2014). Visible light communications (VLC) for ambient assisted living. Wirel. Pers. Commun..

[3] Yamazato T., Takai I., Okada H., Fujii T., Yendo T., Arai S., Andoh M., Harada T., Yasutomi K., Kagawa K. (2014). Image-sensor-based visible light communication for automotive applications. IEEE Commun. Mag..

[4] Uchiyama H., Yoshino M., Saito H., Nakagawa M., Haruyama S., Kakehashi T., Nagamoto N. Photogrammetric system using visible light communication. Proceedings of the 34th Annual Conference of IEEE Industrial Electronics (IECON).

[5] Mikami H., Kakehashi T., Nagamoto N., Nakagomi M., Takeomi Y. (2011). Practical Applications of 3D Positioning Systemusing Visible Light Communication.

[6] Tanaka T., Haruyama S. New position detection method using image sensor and visible light LEDs. Proceedings of the 2nd International Conference on Machine Vision (ICMV).

[7] Nakazawa Y., Makino H., Nishimori K., Wakatsuki D., Komagata H. Indoor positioning using a high-speed, fish-eye lens-equipped camera in visible light communication. Proceedings of the 2013 International Conference on Indoor Positioning and Indoor Navigation (IPIN).

[8] Nakazawa Y., Makino H., Nishimori K., Wakatsuki D., Komagata H. High-speed, fish-eye lens-equipped camera based indoor positioning using visible light communication. Proceedings of the 2015 International Conference on Indoor Positioning and Indoor Navigation (IPIN).

[9] Nakajima M., Haruyama S. (2013). New indoor navigation system for visually impaired people using visible light communication. EURASIP J. Wirel. Commun. Netw..

[10] Nitta T., Mimura A., Harashima H. (2002). Virtual Shadows in Mixed Reality Environment Using Flashlight-Like Devices. Trans. Virtual Real. Soc. Jpn..

[11] Nii H., Hashimoto Y., Sugimoto M., Inami M. (2007). Optical interface using LED array projector. Trans. Virtual Real. Soc. Jpn..

[12] Kimura S., Kitamura M., Naemura T. EmiTable: A tabletop surface pervaded with imperceptible metadata. Proceedings of the 2nd Annual IEEE International Workshop on Horizontal Interactive Human-Computer Systems (TABLETOP ‘07).

[13] Kato Y., Fukasawa N., Naemura T. iPvlc: Pixel-level visible light communication for smart mobile devices. Proceedings of the ACM SIGGRAPH Posters (SIGGRAPH ‘11).

[14] Zhou L., Fukushima S., Naemura T. Dynamically reconfigurable framework for pixel-level visible light communication projector. Proceedings of the Volume 8979, Emerging Digital Micromirror Device Based Systems and Applications VI 89790J.

[15] Hiraki T., Fukushima S., Naemura T. Sensible shadow: Tactile feedback from your own shadow. Proceedings of the Therapeutic 7th Augmented Human International Conference.

[16] Kodama M., Haruyama S. Accurate location service using DMD projector. Proceedings of the 4th International Conference on Serviceology (ICServ ’16).

[17] Kodama M., Haruyama S. Visible light communication using two different polarized DMD projectors for seamless location services. Proceedings of the Fifth International Conference on Network, Communication and Computing (ICNCC).

[18] Kodama M., Haruyama S. (2017). A Fine-grained visible light communication position detection system embedded in one-colored light using DMD projector. Mob. Inf. Syst..

[19] Saito T., Haruyama S., Nakagawa M. (2007). A Study for flicker on Visible Light Communication. IEICE Tech. Rep..

[20] Ishikawa S., Nakagawa M., Haruyama S., Ishikawa S. (2008). Reduction of Flicker by Coding and Modulation for Visible-Light Communication. IEICE Tech. Rep..

[21] Kodama M., Haruyama S. Pulse width modulated visible light communication using digital micro-mirror device projector for voice information guidance system. Proceedings of the 2019 IEEE 9th Annual Computing and Communication Workshop and Conference (IEEE CCWC).

[22] Pascual C., Zukui Song Z., Krein P.T., Sarwate D.V., Midya P., Roeckner W.J. (2003). High-fidelity PWM inverter for digital audio amplification: Spectral analysis, real-time DSP implementation, and results. IEEE Trans. Power Electron..

[23] Inose H., Yasuda Y., Murakami J. (1962). A telemetering system by code modulation −∆ · Σ modulation. IRE Trans. Space Electron. Telem..

[24] Inose H., Yasuda Y. (1963). A unity bit coding method by negative feedback. Proc. IEEE.

[25] Temes G.C., Schreier A. (2005). Understanding Delta-Sigma Data Converters.

[26] Traunmüller H., Eriksson A. The Frequency Range of the Voice Fundamental in the Speech of Male and Female Adults. https://www2.ling.su.se/staff/hartmut/f0_m&f.pdf.

[27] Texas Instruments DLP7000 DLP 0.7XGA 2xLVDS Type-A DMD-TI.com[online]. http://www.ti.com/product/DLP7000/.

